# Epidermal growth factor receptor immunohistochemistry: new opportunities in metastatic colorectal cancer

**DOI:** 10.1186/s12967-015-0531-z

**Published:** 2015-07-07

**Authors:** Ryan A Hutchinson, Richard A Adams, Darragh G McArt, Manuel Salto-Tellez, Bharat Jasani, Peter W Hamilton

**Affiliations:** Centre for Cancer Research and Cell Biology, Queen’s University Belfast, 97 Lisburn Road, Belfast, BT9 7AE Northern Ireland, UK; Institute of Cancer and Genetics, Cardiff University School of Medicine, Institute of Medical Genetics Building, Heath Park, Cardiff, CF14 4XN UK; Waring Laboratory, Department of Pathology, Centre for Translational Pathology, University of Melbourne, Parkville, 3010 VIC Australia; Department of Biomedical Sciences, Nazarbayev University School of Medicine, Astana, 010000 Kazakhstan

**Keywords:** Epidermal growth factor receptor, Immunohistochemistry, Personalised medicine, Heterogeneity, Metastatic colorectal cancer, Image analysis, Localisation

## Abstract

The treatment of cancer is becoming more precise, targeting specific oncogenic drivers with targeted molecular therapies. The epidermal growth factor receptor has been found to be over-expressed in a multitude of solid tumours. Immunohistochemistry is widely used in the fields of diagnostic and personalised medicine to localise and visualise disease specific proteins. To date the clinical utility of epidermal growth factor receptor immunohistochemistry in determining monoclonal antibody efficacy has remained somewhat inconclusive. The lack of an agreed reproducible scoring criteria for epidermal growth factor receptor immunohistochemistry has, in various clinical trials yielded conflicting results as to the use of epidermal growth factor receptor immunohistochemistry assay as a companion diagnostic. This has resulted in this test being removed from the licence for the drug panitumumab and not performed in clinical practice for cetuximab. In this review we explore the reasons behind this with a particular emphasis on colorectal cancer, and to suggest a way of resolving the situation through improving the precision of epidermal growth factor receptor immunohistochemistry with quantitative image analysis of digitised images complemented with companion molecular morphological techniques such as in situ hybridisation and section based gene mutation analysis.

## Background

Personalised cancer medicine, depends upon and requires a detailed analysis of both immunohistochemical and molecular therapeutic targets. Over the last decade, emphasis has shifted from empirical treatment of patients to a biomarker-led, precision approach. Scientific discoveries in carcinogenesis, particularly within the field of molecular pathology, have shaped the personalised medicine paradigm [1–4]. The epidermal growth factor receptor (EGFR) is expressed on the surface of cells of epithelial, mesenchymal and neuronal origin, with an expression range of up to 100,000 receptors per cell. This overexpression sequentially leads to tumour promoting properties such as increased proliferation, evasion of apoptosis and survival [5–9]. Aberrant over-expression of this biomarker has been a widely investigated therapeutic target in a range of solid tumours including colorectal cancer (CRC) with different anti-EGFR therapies being considered.

Following the application of immunohistochemistry (IHC) within the field of diagnostic histopathology in late 1970’s, this method has remained popular for detecting and visualising cellular proteins in tissue samples where it can be used for both clinical diagnosis and classification of tumours [10, 11], including the assessment of EGFR over-expression. The conventional, visual assessment of protein expression within a tissue microarray or whole tissue section involves subjective scoring of the tumour cells and normal cells according to the intensity and the distribution of the stain. However, this subjective classification system does not sufficiently define the cellular and sub-cellular categories, and where the immunohistochemical heterogeneity of cells is not adequately taken into account [12]. For example, classifying the tumour based only on the highest intensity—the so-called ‘hot spot’ method, allocates little significance to regions that have stained with a lower intensity. The heterogeneity of staining observed within tumours and its interpretative complications have been highlighted in a number of recent seminal papers [13–19].

Modern biomarker focused clinical trials must aim to identify reliable prognostic and/or predictive biomarkers for patient stratification [20, 21]. However, variation in IHC methodology, lack of a standardised scoring criteria for EGFR and tissue heterogeneity introduce a range of variables which impact the reliable application of EGFR IHC as a method to determine treatment efficacy [22].

## Immunohistochemical variability in EGFR detection

In both the clinical and translational research setting, protein quantification and visualisation are important [10, 23]. The most common method of assessing protein expression in cancerous cells is immunohistochemistry, with other methodologies such as next generation sequencing (NGS) also being used to detect appropriate targets in specific cancer types [24]. The advantage of using IHC is that this method is fast, cost effective; available within all routine diagnostic laboratories and retains tissue context. However, it has the major disadvantage of being qualitative or at best semi-quantitative visual scoring with inherent inter- and intra-observer variability [25–27]. The limited predictive utility of EGFR expression for the benefit of EGFR monoclonal antibody therapy may be due to a myriad of pre-analytical variables [28–30]. Chromogenic intensity of immunohistochemical assays have been shown to be affected by the type of fixative and the duration of tissue fixation, duration of storage and the conditions of the immunohistochemistry methodology [28, 30–32]. Although EGFR IHC is not used in current clinical practice for selecting patients for cetuximab treatment, there are no universally standardised methodological guidelines [20, 21] unlike HER2 interpretation and other IHC markers [33–37]. This in turn has made both the reproducibility and interpretation of EGFR results somewhat difficult. Studies have found that some EGFR IHC results in various cancer types are dependent on the type of antibody used [11, 38]. Currently, the DakoCytomation^®^ EGFR PharmDx^®^ kit [K1492, Clone 218-C9, Dako, Glostrup, Denmark] remains the only FDA approved method for EGFR detection. Despite the pitfalls in EGFR IHC, EGFR remains one of the most commonly investigated cancer biomarkers due to its oncogenic role in various tumour types [11, 39, 40]. The clinical utility of EGFR expression detection by IHC in colorectal cancer is still somewhat inconclusive, with numerous studies failing to demonstrate a predictive and/or prognostic role for EGFR IHC as a companion diagnostic test for cetuximab [20, 41–43].

## EGFR expression does not correlate with response to anti-EGFR therapies?

**Figure 1:**
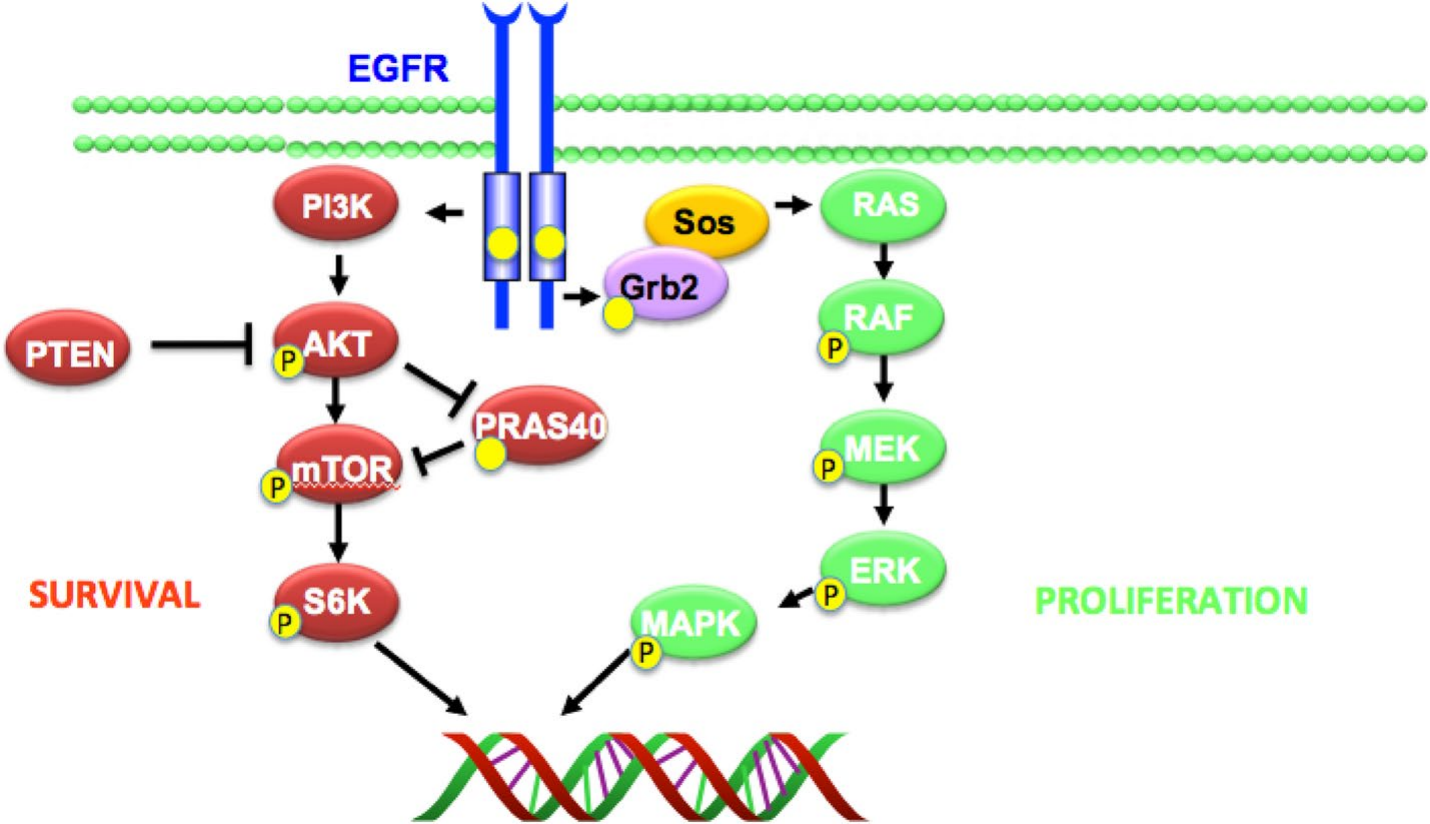
Schematic of the epidermal growth factor receptor and downstream pathways.

**Figure 2:**
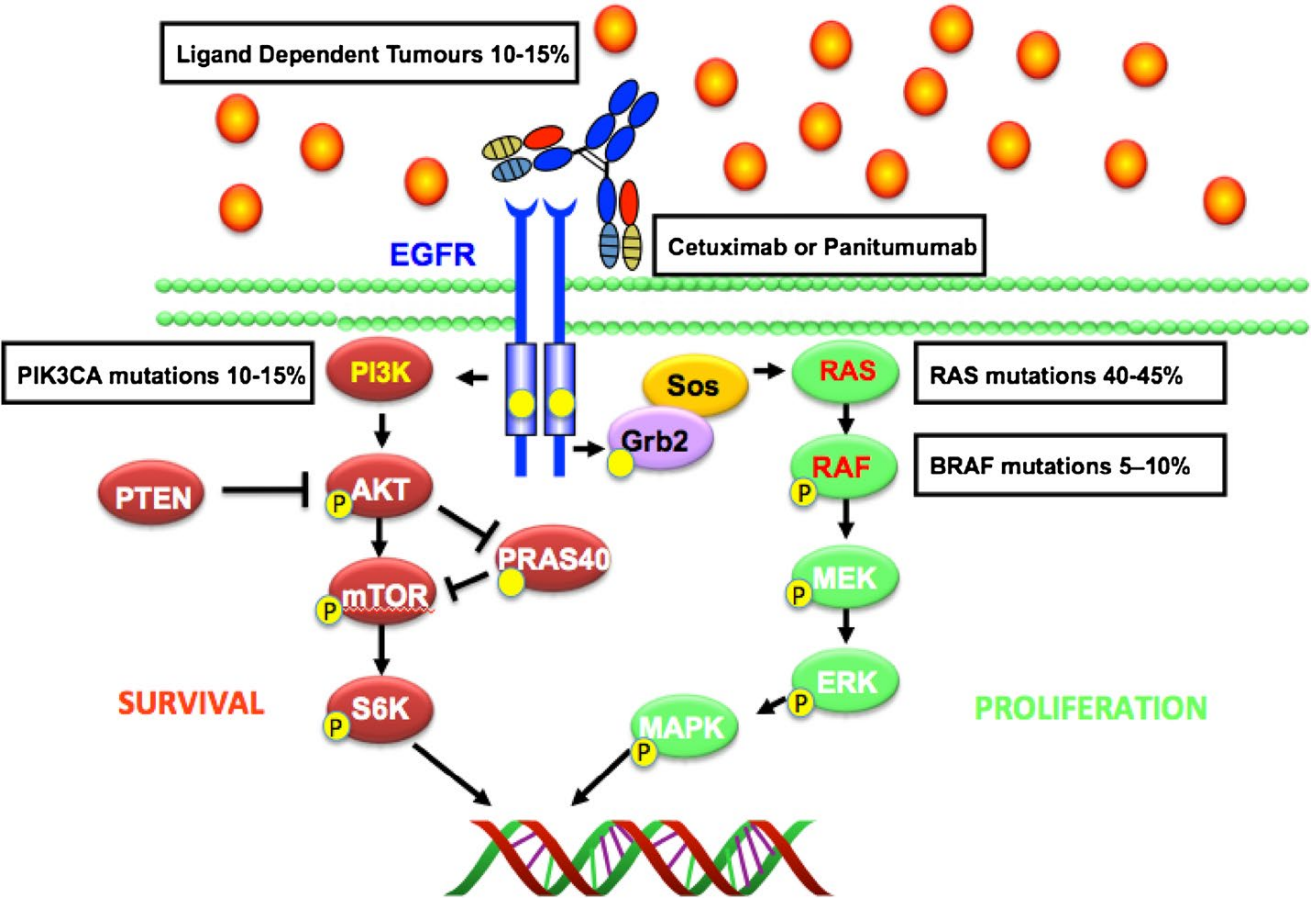
Inhibition of EGFR signalling can be achieved with the use monoclonal antibodies cetuximab and panitumumab in RAS wild type patients.

The EGFR and downstream components of the pathway have an integral role in tumorigenesis by means of regulating proliferation, angiogenesis and metastasis [7, 8, 39] (Figure 1). EGFR inhibition can be achieved by using two classes of drugs, tyrosine kinase inhibitors or monoclonal antibodies [44]. Cetuximab and panitumumab are monoclonal antibodies that bind specifically to both EGFR homodimers and its heterodimers [39, 40, 44]. Cetuximab is an IgG1 chimerised, monoclonal antibody containing 34% mouse protein, which binds specifically to EGFR and its heterodimers [45] (Figure 2). Panitumumab is a fully humanised IgG2 antibody and has been found to have less hypersensitivity reactions compared to cetuximab [46]. KRAS and NRAS mutations have been identified as negative predictive markers for cetuximab and panitumumab efficacy in colorectal cancer [47–50] (Figure 2). This mutation is now used in current clinical practice to stratify patients eligible for cetuximab administration. However, recent studies identified that tumours with a KRAS wild type (KRAS WT) and positive EGFR expression as measured by IHC was not predictive of anti-EGFR efficacy [20, 42, 50, 51].

The use of cetuximab and panitumumab as both a single agent and in combination with chemotherapy has shown efficacy in several studies [20, 45, 52–54]. Results published from the COIN and CRYSTAL clinical trials, and more recent scientific literature seems to suggest irinotecan ±5-fluorouracil as preferred chemotherapy partners to be used with cetuximab [20, 42, 55–58]. A recent meta-analysis by Vale et al. [59] suggest that the differences in the effect may be partially explained by the use of oxaliplatin-based or irinotecan-based chemotherapy. Chung et al. reported that 25% of patients who were found to be EGFR negative demonstrated a complete response to anti-EGFR therapy, the National Cancer Institute of Canada Clinical Trials Group CO.17 Trial enrolled patients on the basis of EGFR IHC positivity; however, there was no correlation between expression and response [20, 41, 42, 56, 60, 61]. Also the conventional IHC antibodies used do not reliably detect EGFR mutations [62, 63], or differentiate between high affinity and low affinity binding receptors [64], which may in turn account for the lack of correlation between EGFR expression and response to EGFR targeting therapies. From the reports of EGFR negative patients benefiting from cetuximab therapy, it may be hypothesised that these patients may indeed have a high ratio of low affinity to high affinity binding receptors and/or EGFR variants within the tumour [60, 61]. Another possible reason for an EGFR IHC negative tumour responding to the treatment response may be heterogeneous overexpression of EGFR in such cases similar to apparently HER2 negative breast cancer cases showing response to Herceptin treatment [65, 66]. The opinion within the clinical setting is that the current recommendations for EGFR detection and determining cetuximab efficacy in colorectal cancer from these are not ‘fit for purpose’.

## Potential for molecular methods as companion confirmatory diagnostic tests for EGFR IHC

Determining EGFR gene amplification status and gene copy number (*GCN*) using fluorescence in situ hybridisation (FISH) and silver enhanced in situ hybridisation (SISH) has been shown to be extremely useful in determining efficacy of some classes of anti-EGFR therapies [67–69]. Major advantages of using SISH rather than FISH is that SISH can be assessed using a bright-field microscope and probe signal remains stable in storage unlike fluorescent probes [69–72]. Attempts to standardise a method for EGFR FISH yielded inconclusive results, with inter-laboratory variability of scoring cut-offs [73, 74]. Similar to that of KRAS mutational status, patients who have been found to have a low EGFR G*CN* are unlikely to respond to treatment with either cetuximab or panitumumab [69, 70, 74, 75]. A study carried out by Personeni et al. found that EGFR G*CN* could be used to predict the outcome after treatment with cetuximab in colorectal cancer patients and was able to predict response and overall survival independent of KRAS status [76]. However, like many other EGFR studies in colorectal cancer the cut-offs used in the complete patient cohort did not perform as well as in the training set from which they were derived and the authors recommended that their cut-offs should not be used as part of any decision making process [76]. Algars et al. [70] demonstrated a clinical benefit from anti-EGFR therapy using EGFR gene copy number, from regions of high EGFR expression in KRAS WT patients to determine response to targeted therapies. This was different from the method used by Personeni et al. [76] in a molecularly unselected population. The aspect of non-molecularly defined cohort may account for the inability of Personeni et al. [70, 76] to standardise reproducible SISH cut-offs for the clinical setting. In relation to determining the regions of high EGFR expression, it is important to note that the antibodies used were not from the FDA approved PharmDx™ assay.

## The role of sub-cellular localisation of epidermal growth factor receptor

EGFR protein expression in colorectal cancer has been widely reported as membranous; however, numerous studies have noted the expression of EGFR within the cytoplasm of tumoural cells [77–79]. Unlike HER2, positive expression of EGFR is not predictive of response to anti-EGFR therapies, however, overexpression has however been linked to a poorer prognosis in colorectal cancer [20, 42, 80–82]. Upon interaction with a ligand the EGFR is internalised which initiates a complex signalling cascade and is degraded in the lysosomal compartment within the cytoplasm [7, 8, 83–85]. In previous studies in pancreatic and thyroid cancer, cytoplasmic expression of EGFR has been linked to a poor prognosis [77–79, 86, 87]. These studies suggest that the cellular localisation of EGFR depend on tumour stage and cancer context and may have significant clinicopathological value particularly in those patients treated with cetuximab with predictive or prognostic utility. Although Chung and colleagues [60] demonstrated that patients benefited from the cetuximab in the absence of membranous EGFR staining, what was not reported was whether any patients exhibited cytoplasmic EGFR staining.

Furthermore, the cytoplasmic localisation of EGFR in both RAS wild type and mutant metastatic colorectal cancer may confer an aggressive phenotype with these tumour cells having an altered intracellular metabolism and may be indicative of tumour cell population having undergone epithelial to mesenchymal transition [88, 89]. Additionally KRAS mutations are known to have different phenotypes with mutations in codon 13 shown to benefit from the addition of cetuximab. KRAS mutations can signal through the RAF-MEK-ERK MAPK pathway or the PI3K-AKT-mTOR pathway, suggesting that cytoplasmic localisation depending on KRAS mutant isoform may have predictive and prognostic utility in RAS mutant colorectal cancers [90–92].

## Extracellular and intracellular mechanisms as predictive markers for anti-EGFR therapies in colorectal cancer

KRAS and NRAS mutations are established negative predictive markers for cetuximab [20, 50, 53, 56–58, 93], and account for approximately 50% of all mutations in colorectal cancer, coupled with other mutations approximately 40% of patients are eligible for anti-EGFR therapies, however, not all eligible patients respond to these therapies. Even with the extensive molecular characterisation of colorectal cancer [94–96], there are few molecular markers implemented in the clinical setting to determine clinical efficacy of anti-EGFR therapies. There are a variety of molecular markers that are still under extensive investigation such as BRAF and PI3KCA mutations to determine their roles in predicting efficacy [1, 4, 57, 58] (Figure 2). The histopathological evaluation of tissue has demonstrated an integral role of the micro-environment in tumoural development and progression. Studies have identified and highlighted the complexity of the crosstalk between tumour cells and host cells such as immune cells, cytokines and blood vessels which modulates tumour progression [97–101]. This observation has been expanded upon by Galon, Pagés and colleagues which led to the publication of seminal papers which defined the *Immunoscore* and has demonstrated the prognostic and predictive significance of immune involvement in colorectal tumours amongst others [100–108]. Automated interpretation of tissue biomarkers to predict response to therapy and future clinical behaviour of a tumour will provide an objective, standardised and reproducible method for tissue biomarker discovery and validation [22, 109].

Metastatic colorectal cancer has one of the poorest 5-year survival rates which emphasises the need for additional predictive and prognostic markers to facilitate efficient and informative patient stratification [110]. There have been eleven ligands identified that are part of the HER family with various receptor specificity, six of these ligands have been found to associate with the epidermal growth factor receptor which induces dimerization and initiation of complex intracellular signalling cascades [7, 8, 111] (Figure 1). Activation of oncogenic signaling through autocrine, paracrine or juxtacrine mechanisms have been shown to have both a predictive effect for response to targeted therapies [112, 113] and as a resistance mechanism through the activation of alternative survival pathways [114, 115] (Figure 2). Gene expression profiling of these ligands have associated epiregulin (EREG) and amphiregulin (AREG) having a role in cetuximab efficacy [112]. An increased mRNA level of either ligand has been shown to be associated with sensitivity to cetuximab monotherapy which has reflected by the longer progression free survival [112, 113, 116]. Tumours with increased levels of both AREG and EREG ligands and a KRAS WT benefited the most from anti-EGFR therapies these findings shows strong predictive value as KRAS and NRAS have strong negative predictive value [47, 48, 56, 112, 113], with a recent publication reporting that patients with KRAS mutant adenocarcinoma of the lung with induced epiregulin expression was associated with an aggressive phenotype [117].

## Beyond total EGFR: determining pathway activation for predicting anti-EGFR response

The presence or absence of total EGFR has been shown to have no predictive utility for anti-EGFR therapies in mCRC [42, 45, 60], thus emphasising the complexity of not just the epidermal growth factor receptor but of the entire EGFR signalling pathway [118]. Ascertaining the activation status of the EGFR pathway may indeed unveil additional EGFR-mediated insights, which has to date eluded clinical practice. However, the challenge facing this paradigm is how to best measure and interpret activation status, what is well known is the plethora of phosphorylation of proteins within the intracellular compartment of cells. Indeed subtle changes in cellular biochemistry can alter the expression pattern of these proteins as well as gene expression profiles therefore providing misleading information as to the activation status of the cellular pathways [30].

The activation status of most signalling pathways can be determined through the measurement of post-translational modifications, such as the phosphorylation status of key amino acids, involved in signal transduction. Phospho-specific antibodies using immunohistochemistry or western blot analysis can be used to measure these. These dynamic post-translational changes can be used demonstrate inhibition of signalling pathways by investigational drugs. The utility of these pathway activation markers and technologies in clinical trials has, however, been limited by the evanescent nature of amino acid phosphorylation which is rapidly reversed by endogenous phosphatases. Extensive studies have shown that the rate of tissue penetration by fixatives, such as formalin, in most solid tumours is too slow to inactivate phosphatases resulting in preservation of phosphorylation only in the outer few millimetres (<5mm) of the tissue sample [119]. The lack of reliable means to collect, store and ship such samples and to reliably preserve and measure these labile events has often failed to generate reproducible clinically translatable results. However, recent translational studies carried out by Chafin et al. [120, 121] identified a method termed “2+2” as a robust method to preserve these phosphorylated proteins.

What has become apparent in precision medicine that no single field can answer the complexity of cancer. A holistic understanding of the tumour genome, phenome, immunome and a myriad of other “ome” related classifications is needed to realize the potential of precision medicine. Genomic stratification of colorectal cancers has identified at least five molecular subtypes of colorectal cancer all of which have various phenotypes and clinical outcomes [122, 123]. Irrespective of EGFR IHC status, a significant proportion of RAS wild type colorectal cancers are resistant to cetuximab, which suggests other EGFR-mediated or related mechanisms, contribute to this paradox. Thus looking beyond total EGFR is warranted, EGFR can be activated through the auto-phosphorylation of its tyrosine residues that in-turn stimulates a myriad of downstream signaling cascades. Integrating this knowledge into the EGFR paradigm, it is plausible that, if accurately detected, phosphorylated EGFR (pEGFR) may indeed reflect receptor utilization by the specific tumour.

Elegant studies carried out by Prahallad et al. [1] and Corcoran et al. [4] identified that BRAF mutant colorectal cancer cell lines were sensitive to dual inhibition with cetuximab and vemurafenib compared to melanoma cell lines. Indeed as shown by Corcoran and Prahallad inhibition of a single oncogenic mutation in colorectal cancer cells with vemurafenib, the same drug which has shown up to 50% response rates in melanoma, efficacy is not the same [1, 4]. This is indeed an example of how the microenvironment of the specific tissue type can regulate resistance mechanisms to both targeted therapies and chemotherapy treatment. However, by addressing this ‘kinome remodeling’ [124] through the use of combination therapies, efficacy was demonstrated. BRAF mutations are found in up to 60% of all melanomas [125] and up to 10% of colorectal cancers, with BRAF mutant CRCs having a poorer prognosis to both wild type and RAS mutant tumours. Corcoran et al. [4] found that phosphorylated EGFR was overexpressed in over 60% of BRAF mutant colorectal cancers compared to pEGFR in melanoma. Taking these findings in subtype specific context suggests that baseline pEGFR levels may make these tumours more prone to EGFR-mediated resistance suggesting the use of pEGFR as a predictive marker to identify patient populations who may respond to different combination therapy approaches. Thus patients with high tumoural pEGFR levels may indeed respond better to cetuximab and/or erlotinib in combination with vemurafenib, for which there are several on-going clinical trials an example of which is the EViCT trial being conducted in Melbourne (ACTRN12614000486628). Patients with low pEGFR may be better candidates for MEK inhibitors such as trametinib, in combination with vemurafenib which has shown improved clinical efficacy in melanomas compared to those treated with vemurafenib alone [126, 127]. It is plausible that mCRC patients whose tumours are negative for EGFR IHC indeed have elevated pEGFR which may explain why a subgroup of these total EGFR negative patients respond to anti-EGFR therapies.

## Digital image analysis for improving the precision and predictive potential of EGFR IHC

Visual interpretation of immunohistochemistry is a subjective measurement coupled with an arbitrary threshold to differentiate and classify patients into expression categories [128]. In addition to the subjectivity, there are well recognised issues of poor reproducibility in evaluation of tissue structures such as determining viable tumour percentage and tumour cellularity for molecular testing and grading systems such as Gleason grading in prostate cancer [129–131]. The increased use of digital slides and whole slide imaging in the last decade has ushered in an exciting era of computer-aided histopathology, with image analysis approaches providing a powerful companion tool for the extraction of quantitative data from digital images in a robust and reproducible manner [132]. Not only does this quantitative, multi-parametric data enable clinical correlations but also offers the ability to visualise quantitative tumoural phenotypes and provide deeper insights into the biological characteristics of tissue specimens. As stated in the preceding sections, immunohistochemistry is an extremely important tool for the identification of single disease-related protein biomarkers [10]. Although the advantages of immunohistochemistry in biomarker discovery and validation are clear, the accompanying issues of inherent subjectivity and poor reproducibility of chromogenic interpretation of routine biomarkers and tissue architecture even by experienced pathologists are well described [109, 128–130]. Image analysis can significantly improve EGFR IHC evaluation through the quantification of expression within an automatically detected tumoural regions, providing an objective histological score. In recent guidelines published for the interpretation of biomarkers in breast cancer, the American Society of Clinical Oncology and the College of American Pathologist’s recommended quantification of ER, PR and HER2 by image analysis and in recent years some commercial image analysis algorithms have been approved for the evaluation of these markers [34, 35]. Riley et al. [133] used a novel quantitative approach to investigate the co-localisation of biomarkers within the cytoplasm and nucleus in NSCLC within both tumoural and stromal tissue, highlighting prognostic clinical insights as to the expression of these biomarkers in various histological subtypes, which may benefit from targeted therapies. Of recent promise and excitement is the development of an automated approach by Galon and colleagues to quantify the type, density and localisation of immune cells in cancer, which has been shown to have prognostic utility across multiple cancer types [101, 102, 106]. A comprehensive review on the role of digital pathology and image analysis in tissue biomarker research has recently been published by our group [109]. Quantitative studies of EGFR IHC are underway by a number of groups which may throw new light the role of EGFR in predicting response to therapy. These studies and the integration of ‘big data’ obtained from quantitative image analysis are likely to be important in defining a specific cancer phenome on a patient by patient basis, establishing new patient signatures and helping deliver the personalised medicine promise of ‘the right drug, for the right patient’ [134–139].

## Conclusion

EGFR over-expression in colorectal cancer as determined by immunohistochemistry has led to initial clinical trials investigating patient selection for cetuximab and panitumumab therapies and shown promising results [41, 45]. However, what is now apparent is that patients can benefit from the addition of cetuximab in the absence of positive EGFR immunohistochemistry [20, 42, 46, 51, 60]. The impact of both intra- and inter-tumoural heterogeneity on therapeutic response, and an effective screening methodology has yet to be clinically implemented [140–142]. Additionally it is important to acknowledge that pre-analytical variables alone do not solely contribute to our inability to predict responsiveness to anti-EGFR therapy using tissue-based EGFR analysis. Indeed the complexity of the EGFR pathway as highlighted by a multitude of publications in recent years merits more in-depth studies in relation to this pathway.

The tumour tissue investigated for EGFR expression is usually the primary tumour sample, however, this may not reflect the molecular landscape and immunohistochemical profile of the metastatic site [13, 57, 58, 143, 144]. Studies by Chung and other authors have proposed that the expression of EGFR may vary within cancer types due to the ratio of low to high affinity binding receptors, which are not differentiated between by the commonly used EGFR assays [60, 62, 145–147]. Rather than relying upon an IHC method alone for determining patients who will benefit from cetuximab a more reliable approach based on a two-pronged strategy utilising a combination of EGFR IHC and EGFR SISH could be utilised to determine the EGFR status. Using such a combined approach it has been possible to identify clinically beneficial therapeutic cohorts of patients based on highest EGFR protein expression and assessing the gene copy number specifically within this region [70]. There is therefore a case to develop an assay for EGFR similar to that of the Dual *ISH* approach in HER2 [148]. In addition to the benefits of the dual method there is also the technically feasible scope for assessing the two independent molecular markers of the EGFR status on a single tissue section making the overall analysis more precise and reproducible.

Digital pathology and image analysis may indeed serve as companion prognostic and or predictive tools in personalised medicine [109], with some companies receiving FDA clearance for algorithms evaluating tissue biomarkers such as HER2, ER, PR and Ki67. For EGFR, quantitative analysis of cell membrane expression may not be sufficient for patient stratification and selection for anti-EGFR therapy, and more precise localisation of the biomarker may be key. Whilst difficult to achieve this using visual scoring, this becomes entirely feasible using computer-aided image analysis. In the current immunohistochemical interpretation guidelines for EGFR and HER2, cytoplasmic localisation is not included. Expression of immunohistochemical markers must be evaluated depending on cellular context and in a cancer specific manner; the prognostic utility of sub-cellular localisation of biomarkers is exemplified by beta-catenin expression and localisation in solid tumours [149]. Molecular interactions are both intracellular and extracellular, and so it is a reasonable assumption that the internalisation and cytoplasmic accumulation of EGFR may indeed alter tumour cell morphology, metabolism and confer an invasive cellular phenotype [88, 89]. Adopting a novel digital pathology based approach for the evaluation of EGFR IHC expression in both the cytoplasm and membrane may elucidate clinically beneficial subgroups that could benefit from the addition of targeted therapies. The objective and reproducible approach of image analysis in tissue biomarker evaluation could initiate a paradigm shift in the way in which we evaluate tissue biomarkers for patient stratification and personalised therapy regimens and indeed the way in which cellular immunohistochemistry is reported [22].

The TCGA consortium reported the frequencies of up-regulation of other HER family members in colorectal cancer which provides scientific and clinical rationale for combination therapies to block different members [1, 4, 96, 150, 151]. Furthermore, as demonstrated by multiple authors through the action of EGFR ligands, tumours do not have to express the EGFR to elicit a response to EGFR-targeted therapies, implying that cetuximab resistance and indeed response in specific cohorts may be governed by ligand-dependent and/or ligand-independent mechanisms [112, 113]. Spano et al. reported that immunohistochemical expression of EGFR was stage-dependent which may suggest that membranous expression of EGFR may not have a predictive utility for monoclonal antibody therapy in the metastatic setting as demonstrated in previous clinical trials however, may indeed have clinical utility in the adjuvant or neo-adjuvant setting [20, 42, 45, 80]. Furthermore, using either total EGFR IHC and/or pEGFR IHC in a subtype specific context may enable more effective patient stratification approaches in CRC as demonstrated by Prahallad et al. [1] and Corcoran et al. [4].

Before we abandon EGFR IHC as an unreliable companion diagnostic for patient selection, a detailed investigation is necessary to include quantitative image analysis of aberrant EGFR expression within the membrane and cytoplasmic compartments, combined with ligand expression, molecular analysis of genetic abnormalities such as gene copy number variation and mutational status. This will provide definitive insight into EGFR as a tissue-based biomarker in patients with colorectal cancer and other malignancies.

## Abbreviations

EGFR: epidermal growth factor receptor
IHC: immunohistochemistry
CRC: colorectal cancer
GCN: gene copy number
AREG: amphiregulin
EREG: epiregulin
TCGA: the cancer genome atlas
ER: oestrogen receptor
PR: progesterone receptor
pEGFR: phosphorylated EGFR
HER2: human epidermal growth factor receptor 2
ISH: in situ hybridisation
SISH: silver in situ hybridisation
FDA: food and drug administration
